# Pharmacy role in the proMotion of continencE (PRIME): a co-production study to develop a pharmacy bladder and bowel service

**DOI:** 10.1186/s12913-026-15123-8

**Published:** 2026-07-24

**Authors:** Nikki Cotterill, Alan Uren, Ade Williams, David Chandler, Hugh McLeod, Shoba Dawson, Margaret Watson

**Affiliations:** 1 School of Health and Social Wellbeing, College of Health, Science and Society, University of the West of England, UWE Bristol, Blackberry Hill BS16 1DD UK; 2 Bristol Urological Institute, Southmead Hospital, Bristol, UK; 3 Bedminster Pharmacy, Bristol, UK; 4 Population Health Sciences, Bristol Medical School, Bristol Medical School, University of Bristol, Bristol, UK and National Institute for Health and Care Research Applied Research Collaboration West (NIHR ARC West) at University Hospitals Bristol and Weston NHS Foundation Trust, Bristol, UK; 5 Centre for Academic Primary Care, Bristol Medical School, University of Bristol, Bristol, UK; 6 Watson Research and Training Limited, Aberdeen, AB15 8FL UK; 7 Strathclyde Institute of Pharmacy and Biomedical Sciences, University of Strathclyde, 160 Cathedral Street, Glasgow, G4 0RE UK; 8 Patient/Public contributor, Bristol, United Kingdom

**Keywords:** Community pharmacy, Continence promotion, Co-production, Faecal incontinence, Health promotion, Urinary incontinence

## Abstract

**Background:**

Urinary and faecal incontinence affects millions. Community pharmacies provide local community healthcare highlighting opportunity for innovation in continence promotion. The Pharmacy Role In the proMotion of continencE (PRIME) study was conducted to investigate development of a Pharmacy Bladder and Bowel Service (PBBS) incorporating key stakeholder engagement.

**Methods:**

A UK online survey, employing convenience sampling of community pharmacy staff identified current practice, and barriers and facilitators to PBBS development, between July and November 2022. These findings were incorporated into an evidence brief supported by findings from earlier PRIME sub-studies (systematic literature review and qualitative interviews). The evidence brief guided stakeholder participant workshop discussions using co-production methodology, during April and June 2023, to derive a protocolised service specification for the PBBS, detailing underpinning requirements and scope of service, as the final stage of this study.

**Results:**

Twenty-nine community pharmacy participants completed the survey reporting some provision of lifestyle advice in practice with clear scope for expansion. Subsequently, sixteen participants including Community Pharmacists, Bladder and Bowel Service staff, Service users and healthcare commissioners participated in two workshops. The PPBS service specification outlined three tiers of service provision: tier 1 – awareness raising, education and signposting; tier 2 – assessment and self-help advice; tier 3 – specialist input involving specific treatment strategies. Acknowledged limitations are the pressures and relatively novel nature of pharmacy research which presents challenge in promoting participation.

**Conclusions:**

This is the first evidence-based PBBS service specification, developed to inform future practice. Key requirements include record sharing, advances in training, and reimbursement to enable the PBBS to be viable for community pharmacy provision. The PBBS provides potential for early identification, promotion of continence health, and self-help activation in community settings.

**Supplementary Information:**

The online version contains supplementary material available at https://doi.org/10.1186/s12913-026-15123-8.

## Background

Urinary and faecal incontinence remain a taboo in society, yet they are a significant issue with over 14 million adults experiencing bladder control problems and 6.5 million with bowel control difficulties in the UK [1]. People are often reluctant to disclose symptoms, preventing access to evidence-based strategies that can facilitate improvement [2]. Incontinence is known to impose significant decrement on physical and psychological wellbeing, impacting every aspect of personal, social, relationship and work life.

Incontinence costs the National Health Service (NHS) over £700 million annually and substantial personal costs are also incurred, reported to be £226 million per annum on continence products alone [1].

Previous engagement activity among people with lived experience conducted by the study team has highlighted the need for increased awareness surrounding incontinence and accessibility to interventions to achieve symptom improvement [3–5]. The first line of treatment for urinary or faecal incontinence in National Institute of Health and Care Excellence (NICE) guidance and international consensus guidelines, is conservative management including self-help strategies such as fluid and dietary advice and pelvic floor muscle exercises [2, 6, 7]. A sizeable gap exists between evidence-based interventions and those who can benefit from this knowledge with communicating this identified as a key priority for this clinical area [3].

Pharmacies are ‘closer to home’ than most healthcare services with 89% of the population within a 20-minute walk of a community pharmacy in England, increasing to 99% of the population in areas of highest deprivation [8]. This is pivotal to the priority to address health inequalities [9]. Increasingly community pharmacy is being recognised as an intrinsic component of the healthcare pathway for a range of conditions. The community pharmacy setting provides an opportunity to provide education, treatment and self-help strategies as highlighted above, along with signposting to more specialised support such as Bladder and Bowel Services where required for both patients and carers [10, 11]. Community pharmacies are perceived to be convenient and a less formal environment to GPs or specialist clinics. Hence the initiation of ‘Pharmacy First’ in England, enabling consultation in community pharmacy as the first point of contact for seven common conditions, of which only uncomplicated urinary tract infections has relevance to the Urology speciality [12].

**Fig. 1:**
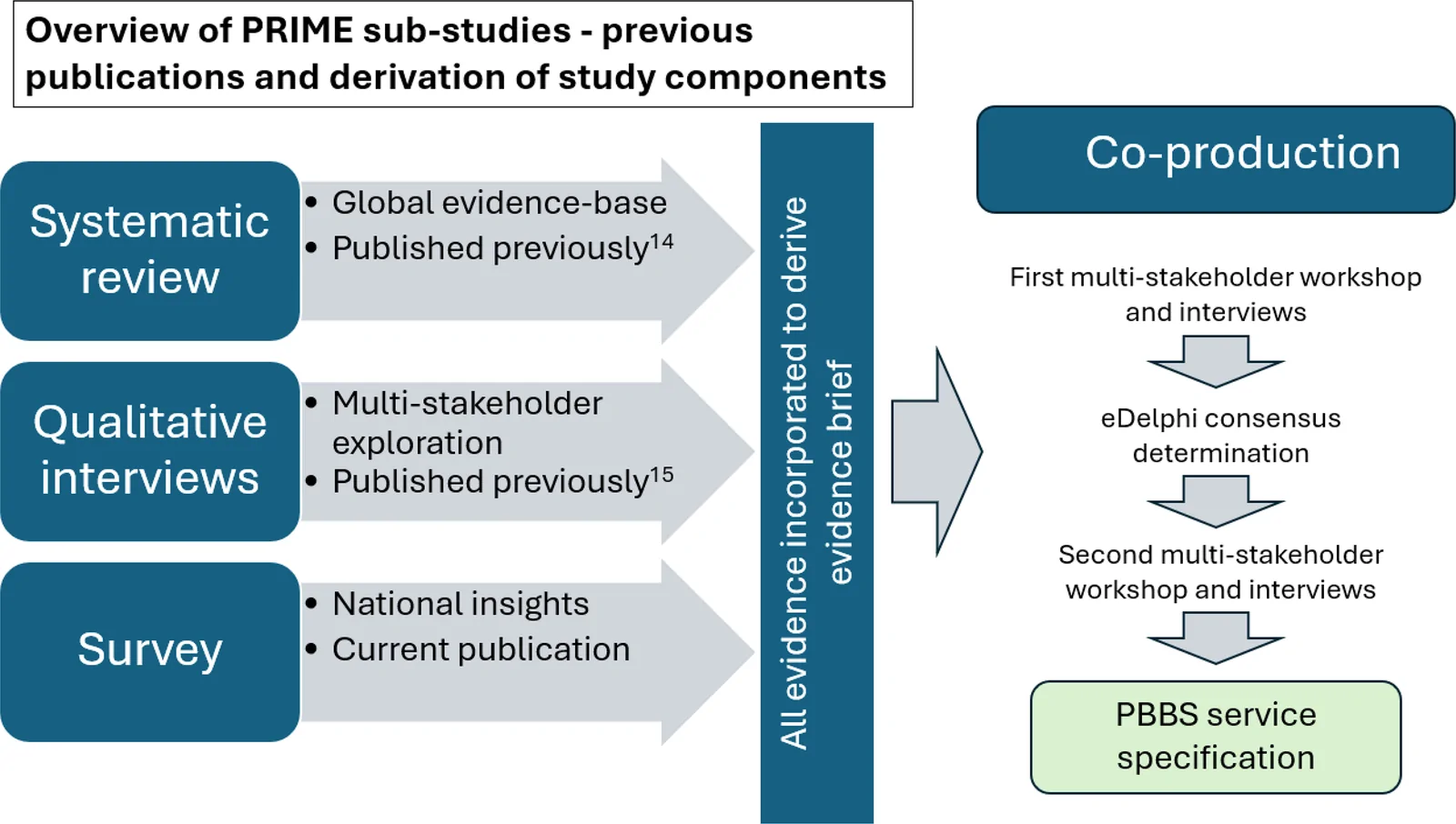
Progression and overview of PRIME study components detailing previously published studies and their contribution to current research activity

There is a paucity of evidence in the literature on continence health promotion in community pharmacy, and there is no definitive model of best practice [13]. This finding was supported through a systematic review of global literature undertaken by the study team as the first component of the Pharmacy Role In the proMotion of incontinencE: PRIME study (NIHR202212), initiated in 2021 to develop a Pharmacy Bladder and Bowel Service (PBBS) framework (Fig. 1) [14]. The second component of the PRIME programme involved qualitative interviews to provide in-depth exploration of the opportunities and potential challenges to a PBBS among key stakeholders [15]. The final two phases of PRIME, to achieve the described objectives, are detailed here:

1. UK online community pharmacy survey to gain insights into existing practice and potential future opportunities for continence promotion in this sector,
2. Co-Production workshops to collaboratively develop the service specification for the PBBS describing the scope of provision in practice.

These sub-studies form the first phase of the Medical Research Council’s (MRC) Framework for Developing and Evaluating Complex Interventions [16]. These studies specifically achieve ‘Development of the intervention’ as outlined in the MRC framework with focus on the key considerations: context, how it will work, stakeholder engagement, key uncertainties, refinement and cost considerations.

## Methods

### Survey of community pharmacists

A cross-sectional online survey ((Qualtrics, Provo, UT) was developed for the study purposes by clinical experts in continence and community pharmacy practice within the project team and through wider professional networks, and informed by literature identified in earlier sub-studies [14, 15]. It was designed to explore existing continence practices and investigate opportunities for continence promotion along with likely barriers that require consideration to enable implementation. Questions and response frames were designed in conjunction to represent real world practice, NICE guidance and facilitate easy completion for participants [17]. The survey also explored previous continence-related training (Appendix 1). National pharmacy networks distributed the survey through newsletters and social media posts including organisations such as the Pharmaceutical Services Negotiating Committee and outreach through sector-wide publications, between July and November 2022. Convenience sampling was employed with repeated posts intended to encourage participation from the eligible audience of all personnel working in community pharmacies. The analysis drew on descriptive statistical techniques, using basic counts and proportions to condense the raw data into an accessible summary that highlighted the principal trends within the sample.

### Co-production workshops

Co-production methodology was identified as the most appropriate approach to this research in particular the core principles of a participatory approach in which professionals, service users, families and communities work in equal and reciprocal partnership to design solutions [18]. The value of a shift from traditional dependency models to co-dependency enabled joint mobilisation of knowledge and lived experience, from service user and provider perspectives, to produce services that are more effective, deliverable and responsive to community needs [19]. Key stakeholders were recruited to participate in workshops and a consensus-determining exercise, between April and June 2023 from four identified groups: service users; community pharmacists; bladder and bowel service healthcare staff; and commissioners within NHS integrated care services. These groups were identified as those with critical perspectives of the proposed new service experience and its provision, along with those providing services that will align with the new PBBS and the funding decision makers at a system level. Purposive sampling was employed for balanced recruitment and to ensure a group size that enabled all participants to contribute effectively. The participant group intended to include increased representation of service users, equal group sizes for community pharmacy and bladder and bowel service personnel, and a smaller number of commissioners, as this reflects the relative involvement of these roles in the health system. In accordance with the methodological approach, workshop attendees were provided an evidence brief informed by findings derived from the previous sub-studies [20] (Appendix 2). Deliberative dialogue was held [21], informed by the evidence brief and a User Journey approach was adopted. The User Journey approach explores a user’s interaction or experience with a service and was used to explore both the considerations of those using and providing the proposed service. The MoSCoW method (Must have, Should have, Could have, Won’t have) [22] is a validated technique that was incorporated to establish the importance of each requirement for the service. This approach enabled all participants to share their reflections on the evidence brief followed by discussion of these reflections. In the spirit of Co-Production, service user participants provided their reflections first followed by healthcare professionals, which is intended to balance power dynamics that can arise. Participants unable to attend the first workshop were followed up in one-to-one interviews. All workshops and interviews were conducted using online video platforms and recorded to enable analysis of the findings. From the discussions held, suggestions were identified for the PBBS and an eDelphi method employed to determine participant consensus as outlined below [23, 24]. Participants were asked to complete a survey comprising the workshop suggestions, to rate which elements should be included in the PBBS service specification on a Likert scale scored from 1 (disagree with inclusion) to 7 (agree with inclusion). Consensus was agreed a-priori to be achieved for any element that reached > 80% agreement (5,6, or 7 on scale) or disagreement (1, 2, or 3 on scale). In the second workshop, these results were presented to the group followed by in-depth discussion of the areas where consensus had not been achieved, to determine the final components for the PBBS service specification.

NHS Health Research Authority approval was secured (22/HRA/2388 and 23/HRA/0586) and University ethics approval was granted by the University of the West of England (HAS.22.06.117 and HAS.23.02.074), for the survey and co-production sub-studies respectively and the study was conducted in accordance with the Declaration of Helsinki.

## Results

### Survey of community pharmacists

Twenty-nine community pharmacy personnel completed the survey as detailed in Table 1, providing insights on existing practice and potential future opportunities for continence promotion in this sector.

**Table 1:** Community pharmacy survey study participant setting characteristics

Category	Participants n (%)
Pharmacy role:	
Pharmacist	22 (76)
Non-pharmacist personnel (e.g. pharmacy technicians, dispensing staff)	7 (24)
Pharmacy type:	
Single owner, independent single outlet	9 (31)
Single owner, independent outlets (2-5 outlets)	3 (10)
Single owner, independent outlets (6+ outlets)	3 (10)
Partnership, independent single outlet	1 (3)
Partnership, independent outlets (2-5 outlets)	3 (10)
Partnership, independent outlets (6+ outlets)	3 (10)
Large chains/multiples	5 (17)
Other	2 (7)
Location:	
England	20 (69)
Scotland	1 (3)
Wales	1 (3)
Unreported	7 (24)
Products available:	
Pharmacy sells urinary incontinence products	26 (90)
Pharmacy sells faecal incontinence products	13 (45)

An approximation of current demand for continence advice in community pharmacy ranged from less than five people per week to 20 or 30 per week, and up to 70 as reported by two participants. Most activity took place in pharmacy premises, with, one response for each of remote provision and care homes.

**Fig. 2:**
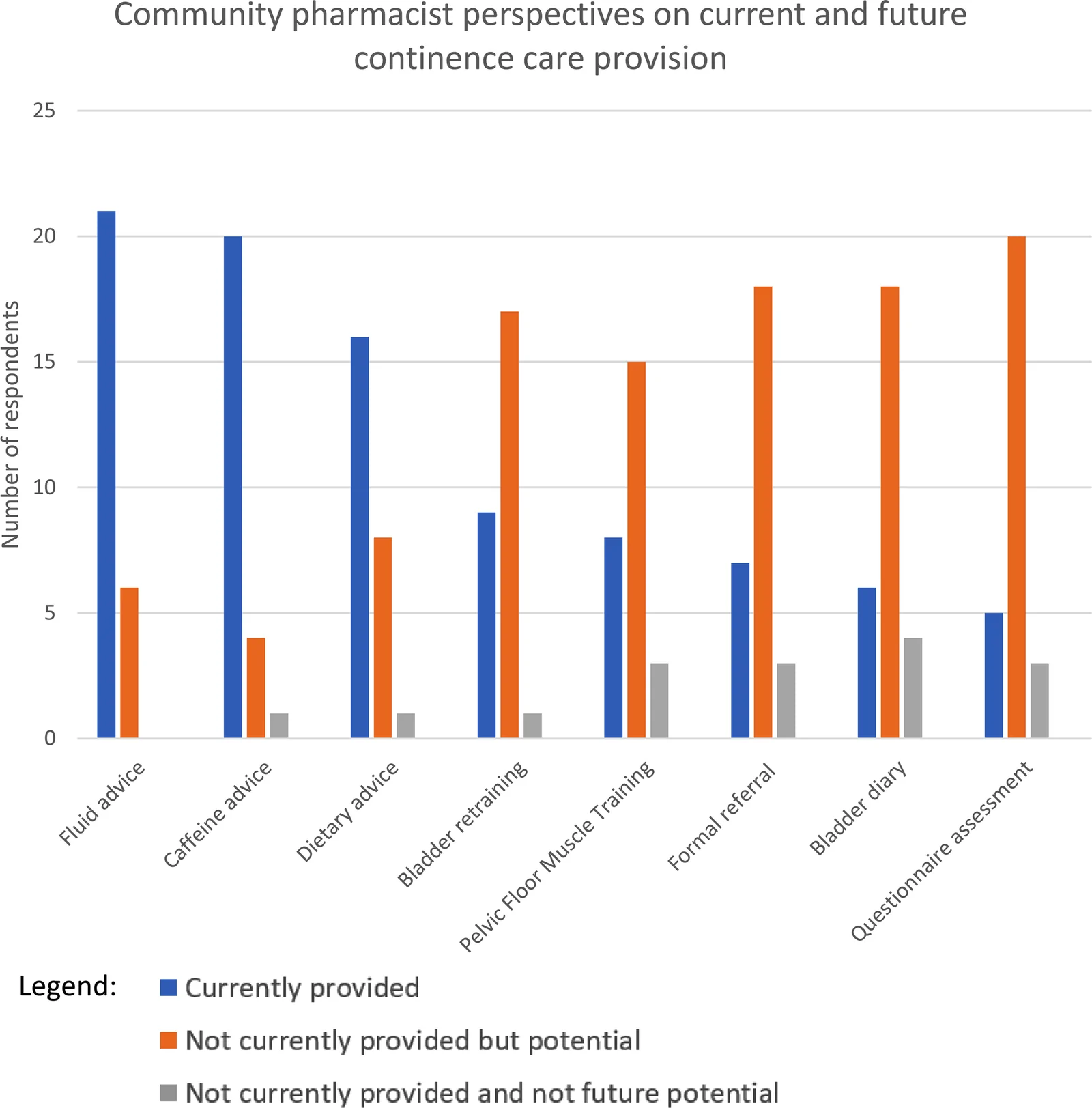
Community pharmacy survey findings of current continence care provision and future potential

Respondents’ current provision of continence care and perception of future provision was investigated (Fig. 2).

With regard to current provision, 69% (*n* = 20) provided advice on caffeine reduction, 72%, (*n* = 21) on fluid management, and 57% (*n* = 16) on dietary factors. Important components of continence promotion indicated in NICE guidance were explored. Activity advocated by NICE that pharmacists do not currently provide but would be prepared to include: 62% (*n* = 18) bladder diary provision; 69% (*n* = 20) symptom assessment questionnaires; 59% (*n* = 17) bladder retraining advice, 54% (*n* = 15) pelvic floor muscle training. Confidence in conducting continence-related activities was also investigated with areas of uncertainty highlighted in making referrals, advice focussed on faecal incontinence, and activity more focussed around what they would be prepared to develop with further training e.g. bladder retraining, pelvic floor muscle training, conducting questionnaire assessments and administering bladder diaries. The majority (83% (*n* = 24)) stated that they would like to be able to do more for people who attended their pharmacy with incontinence.

The key determinants of future continence service provision were access to a private consultation room 83% (*n* = 24), appropriate funding 75% (*n* = 21), adequate staffing 71% (*n* = 20) and access to training 71% (*n* = 20).

Eight respondents reported undertaking training related to incontinence, which was dominated by provision from commercial providers.

### Co-production workshops

Sixteen participants including community pharmacists (*n* = 4), Bladder and Bowel Service staff (*n* = 4), service users (*n* = 6), and healthcare commissioners (*n* = 2) were recruited to attend workshops to collaboratively develop the service specification for the PBBS describing the scope of provision in practice. The evidence brief included an overview of incontinence, community pharmacy and a summary explaining why continence promotion in community pharmacy was being explored. Summaries of findings from the PRIME study components (systematic review, qualitative interviews and survey) were included to enable participants to reflect during the workshops. Two workshops attended by 9 and 8 participants were held, lasting two to three hours each, eight weeks apart. Individual interviews were held separately for those unable to attend and no discernible difference was detected in outputs between the two modalities.

Twelve participants completed the consensus survey between the workshops, achieving representation from each of the key stakeholder groups.

Discussions at the first workshop highlighted four key considerations for the intended PBBS: engagement with a community pharmacy, the consultation, follow-up care, and other aspects such as support and promotion of the service. Within each of these components, suggestions for inclusion were identified which formed the content of the eDelphi survey. Areas of consensus agreement for inclusion are highlighted green (Table 2). Results informed discussions in the second workshop and areas of non-consensus (grey) were the priority focus. Only ‘documentation in paper-based patient records’ was identified as a clear suggestion that should be excluded from the PBBS specification.

**Table 2:**
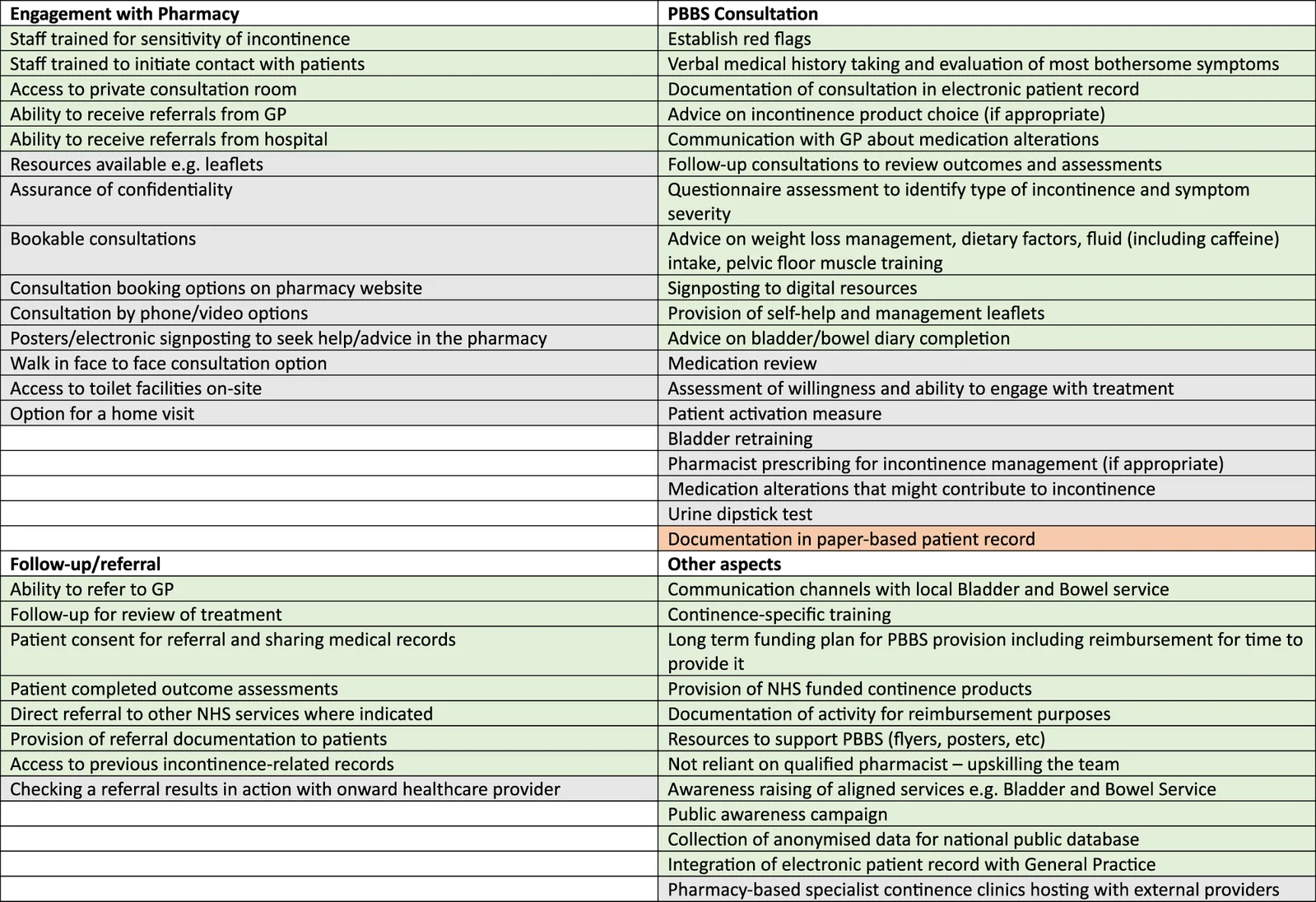
Co-produced suggestions for inclusion in the PBBS and eDelphi consensus survey findings

Legend: Consensus for inclusion achieved – more than 80% of respondents reported ‘agreement’

Consensus not reached

Consensus for exclusion achieved – more than 80% of respondents reported ‘disagreement’

**Fig. 3:**
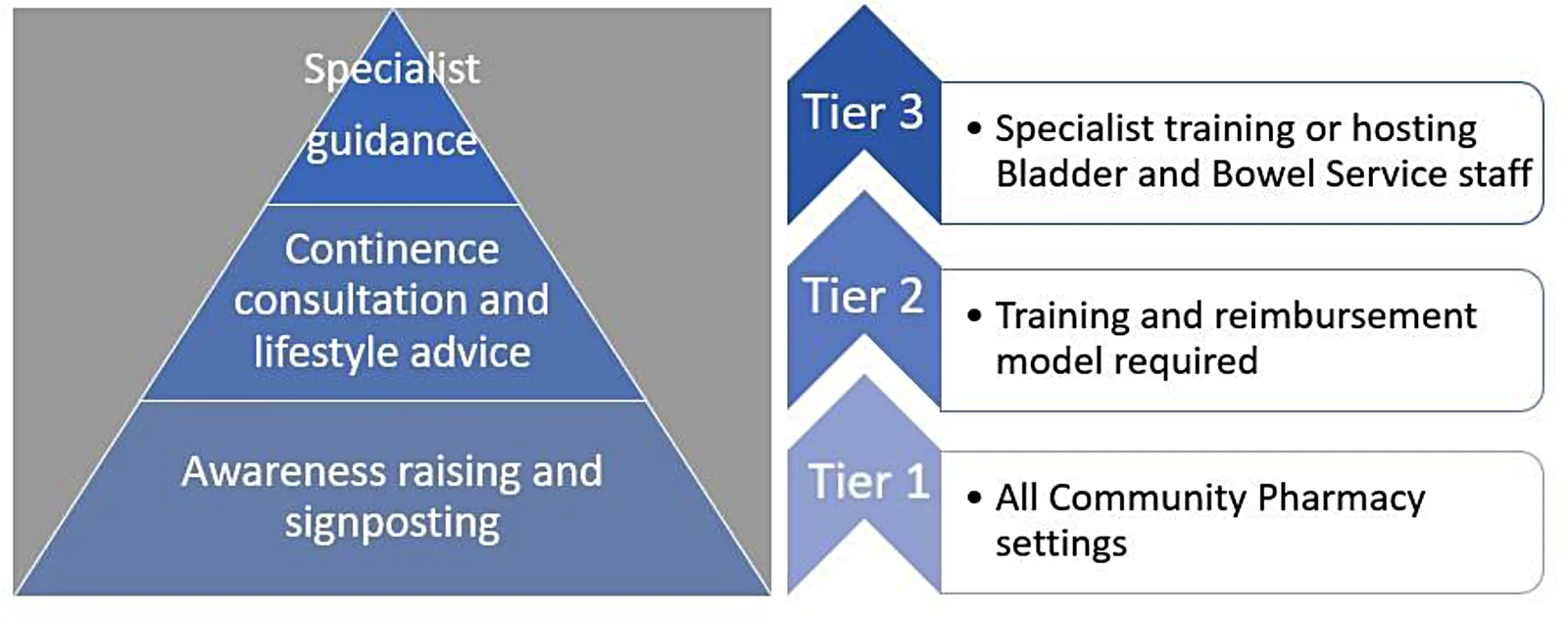
Proposed tiers of pharmacy bladder and bowel service and support requirements

The outstanding areas for discussion centred on toilet accessibility and appointment format; tailoring the scope of assessment/treatment intervention according to local provision capability including staff and facilities; future proofing was repeatedly highlighted avoiding the requirement for outdated paper-based processes; and, managing expectations about integration and onward referral with other services. Points of non-consensus were systematically addressed until agreement was reached among all participants regarding whether each item should be included, excluded, or designated for local contextualisation. Significant variability in facilities and provision capability were highlighted, although it was concluded that most pharmacies now have access to a private consultation space. It was evident that the ability to tailor elements of the PBBS was required according to circumstances and intended provision. The service specification therefore includes areas that can be tailored according to local need, capacity or capability, providing a tiered framework in which each setting could provide some level of service. Capability requirements to enable community pharmacies to provide enhanced levels of service are also identified (Fig. 3).

## Discussion

The PRIME project was conducted to investigate possibilities for continence promotion in community pharmacies. Two previously published PRIME studies (systematic literature review [14] and qualitative stakeholder interviews [15]) informed the two final studies of the PRIME programme, detailed here. The aims of the final studies were to: [1] gain insights into existing pharmacy practice and potential future opportunities for continence promotion in this sector using an online national community pharmacy survey, and [2] collaboratively develop a service specification for the PBBS describing the scope of provision in practice utilising Co-Production to meet the needs of both service users and service providers.

The study completes the initial phase of the MRC complex intervention framework [25], namely identification of the planned intervention, the PBBS, and its evidence-based development in preparation for future evaluation.

There is a paucity of research evidence globally related to continence promotion in the pharmacy setting [14]. The PRIME study provided the opportunity for key stakeholders to consider evidence and the requirements of a PBBS to devise a service that is fit for purpose. Including the perspective of potential PBBS service users and providers, alongside those already providing care in Bladder and Bowel services, and those making service provision decisions through commissioning roles, was a novel approach for community pharmacy service development.

Clear considerations were highlighted including ‘System’ factors such as funding models, ‘Provider’ factors such as training and facilities, and ‘Service User’ perspectives such as privacy and dignity requirements. Addressing these factors is essential to embed PBBSs within the healthcare ecosystem. These factors are included in the draft service specification that will form the basis of an implementation and clinical and cost effectiveness evaluation trial. Within the wider health service landscape these findings support further consideration of the extension of pharmacy services. Given the advent of Pharmacy First during this project, the additional pressure on this sector cannot be overlooked and will be a consideration for national implementation [12].

Since 1st September 2021, medicines for continence care form part of the Community Pharmacy New Medicines adherence supporting service. This is encouraging and recognises the positioning of community pharmacy to influence care related to continence, however, it is focussed on prescribed medication administration. The PBBS service specification is considered a supportive addition to enabling a more holistic approach to continence promotion in this sector. In addition, the positive pharmacy care law, which provides a focus on more community pharmacies being present in areas of highest social deprivation, further supports the intention of the PRIME project to reduce access barriers that can be present in other health services, contributing to health equity [8].

The 2022/23 Health and Social Care Committee report, detailing the evaluation of the Government’s commitments in the area of pharmacy in England was published in July 2023 [26]. The testing of additional services (unrelated to incontinence) was graded ‘requires improvement’, recognising challenges around resource limitations, project management support and information technology systems, which were priorities identified here. Once services are mainstreamed they are funded through the Community Pharmacy Contractual Framework (CPCF). The CPCF confirms community pharmacy’s future as an integral part of the NHS and recognises that an expanded service role is dependent on action to release pharmacist capacity from existing work [11]. This aligns with the findings of our study where appropriate reimbursement, training and infrastructure were identified as key determinants of success for the PBBS. Despite a clear desire to do more for pharmacy users in the community setting, robust financial underpinning is required to enable effective provision in this clinical setting.

The PBBS represents an exciting opportunity to promote bladder and bowel health, informing improved continence education, self-help and health outcomes. Importantly it offers a chance to destigmatise such conversations and improve broader wellbeing and health literacy, including earlier diagnosis of more worrying problems such as cancers [27].

A strength of this study was the inclusion of key stakeholders necessary to provide valuable perspectives on the PBBS, enabling all relevant expertise and experiences to be considered and embedded into its development. It also provided an invaluable opportunity for people with symptoms and healthcare professionals to listen to each others’ accounts to better understand why continence is relevant for community pharmacy. In particular, it was highlighted that lived experiences are critical to the messaging in order to convey the importance of continence promotion in community pharmacy. This highlights the value of embedding patients within service development planning and their role in challenging assumptions about healthcare priorities.

A limitation of the study is that community pharmacy services are stretched and the capacity to participate for personnel in this sector is limited. In the national survey of community pharmacists only twenty nine participants provided responses, which were extremely valuable but represents a small, self-selected sample of this population of over 64,000 qualified pharmacists in the UK [28]. Given the promotion of the survey through multi-media channels of national pharmacy networks, an accurate estimate of the potential participant pool is unknown as we have no certainty regarding reach of the study recruitment materials. Anecdotally, we also know that pressures in community pharmacies during the time of the study may have prevented increased participation. These findings are a small snapshot of activity that may be representative of those with a particular interest in continence promotion and not reflect wider activity in both existing practice and potential for future practice related to continence in community pharmacy. Survey results did reflect the small published evidence base in this field however, providing some reassurance for these outcomes.

## Conclusions

The PRIME study provides an evidence-based structure for the novel PBBS developed from a multi-stakeholder perspective, clearly articulating the requirements for provision within the existing health system. Placing continence promotion and care in the heart of communities could transform early identification and intervention, increase accessibility and address inequalities that are a common feature of formal healthcare pathways. Future research is planned by the study team to establish feasibility of PBBS implementation in practice, and clinical and cost-effectiveness evaluation.

## Supplementary Information

Below is the link to the electronic supplementary material.

Supplementary Material 1

Supplementary Material 2

## Data Availability

The datasets generated and/or analysed during the current study are not publicly available due to the qualitative nature of the content but are available from the corresponding author on reasonable request.
