# Supplementary material for: Pharmacy role in the proMotion of continencE (PRIME): a co-production study to develop a pharmacy bladder and bowel service
Source: BMC Health Serv Res. 2026 Jul 24;26:1445. doi: 10.1186/s12913-026-15123-8 (PMC13689019; doi:10.1186/s12913-026-15123-8)

PRIME Survey

Start of Block: Default Question Block

Q1

Q2 **Survey of incontinence care provision in the community pharmacy sector: current practice and future possibilities**

Q3 Section 1: Participant information sheet

What is this survey about?
We are conducting this survey to explore current practice in community pharmacy regarding the management of individuals who present with bladder and/or bowel incontinence. We are keen to understand pharmacy staff views and needs with regard to the provision of continence care.

Who is this survey for?
The survey should be completed by **one** member of staff working within the community pharmacy. This can be a pharmacist, pharmacy technician, dispensing staff, medicine counter assistant or other: ideally, by the person who knows most about the services and products available from the pharmacy for the management of continence. It is important that we ask staff who could be involved in providing continence care or advice and guidance within community pharmacy. By fully exploring current perspectives we will identify areas to build and improve upon to enhance accessibility to continence care and management.

What will I be asked to do?
Participation in this study is entirely voluntary. Participation requires answering a series of questions separated into different sections. Completion of the survey should take between 10 and 15 minutes. All the answers you provide will be confidential and will be anonymous.

What benefits are there to completing this survey?
Primarily you will be contributing to the knowledge and understanding of continence care in community pharmacy so we can identify ways that it may be improved in the future.

We will also enter all respondents who are happy for their contact details to be recorded, into a prize draw of £50 each for two random winners. This prize can be received by you, your

community pharmacy, or be donated to the Pharmacy Support Charity on your behalf.

How will my data be handled?

The information collected on this survey will be processed by the University of the West of England in accordance with the General Data Protection Regulation as applied, enacted and amended in UK law. The data controller is the University of the West of England.

We will hold your data securely and not make it available to any third party.

1. Why are we collecting this information? We are collecting this information to understand more about current practice in pharmacies, and attitudes and knowledge regarding continence care. Deciding whether or not to complete the survey is entirely your choice.

2. To whom will the information be disclosed/shared and why? All the information collected will be anonymous and individual participants cannot be linked with their responses. The anonymous results will be disseminated through a variety of channels, for example through publication in scientific journals, conference presentations and social media.

3. Where will the information be stored and how secure will it be? The data will be kept on the One Drive platform administered by the University of the West of England and to which only the research team have access. Access will be password protected.

4. How long will the information be kept for and when will it be destroyed? The data will be kept for 24 months after which it will be destroyed.

5. What rights do you have? As data will be anonymous there is no way to identify your personal information in order to access it, alter it, erase it, or otherwise object to its processing. You do have the right to complain to the Information Commissioner's Office (ICO). To find out more or to exercise any of these rights please contact the Data Protection Officer at the University of the West of England. You can do this by writing to the Data Protection Officer, UWE Frenchay Campus, Coldharbour Lane, Bristol, BS16 1QY, or dataprotection@uwe.ac.uk.

6. Who can I ask if I have questions about this study? Dr. Nikki Cotterill, Professor in Continence Care, UWE, is the Chief Investigator for this study and can be contacted on: nikki.cotterill@uwe.ac.uk The study survey is a part of the PRIME Study (Pharmacy Role in the Promotion of Continence) (PRIME), funded by the National Institute for Health Research (NIHR) [Research for Patient Benefit (NIHR202212)].

*

Q4 Section 2: Consent Form
I have read the information above *(Required)*

○ Yes (1)

---

*

Q5 I understand that data I provide will be stored in a secure format, analysed confidentially and that any publication of data will not include personal details *(Required)*

○ Yes (1)

---

*

Q6 I understand the University will process my personal data as described above *(Required)*

○ Yes (1)

---

*

Q7 I understand that I can withdraw at any time during the completion of the study, by exiting the web-page and I do not need to provide a reason for my withdrawal *(Required)*

○ Yes (1)

---

*

Q8 I agree to take part in the study *(Required)*

○ Yes (1)

Q9 **Thank you for agreeing to take part in the survey.**

Q10 Section 3: Current provision of continence care

Throughout the survey, the term 'incontinence' is used to refer to any occurrence of bladder and/or bowel leakage, and 'continence' is the term used to describe the ability to avoid leakage. Anything related to these symptoms should be considered.

Q11 Q1. Which continence products are available to buy from your pharmacy? *(Please tick all that apply).*

- ☐ Incontinence pants (1)
- ☐ Incontinence pads (2)
- ☐ Bed underpads (3)
- ☐ Incontinence sheets (4)
- ☐ Night pads (5)
- ☐ Underwear liners (6)
- ☐ Waterproof pants (7)
- ☐ Bowel incontinence pads (8)
- ☐ Bowel incontinence devices (9)
- ☐ Other (10)

Q12 Q1a. Please provide details if 'other'.

____

Q13 Q2. On average, how many patients/customers come to your pharmacy each week with continence-related needs e.g.asking for advice or buying incontinence products? *(Please enter an approximate number)*

____

Q14 Q3. Where is most of your continence-related activity conducted currently?

- ○ At the pharmacy premises (1)
- ○ Remote support (Online or on the phone) (2)
- ○ Visits to other organisations such as Nursing Homes (3)
- ○ Other (4)

Q15 Q3a. Please provide details if 'other'.

____

Q16 Q4. Continence care is part of my role in this pharmacy.

- ○ Strongly agree (1)
- ○ Agree (2)
- ○ Unsure (3)
- ○ Disagree (4)
- ○ Strongly disagree (5)

Q17 Q5. Which of the following tasks do you consider to be **part of your role** in providing continence care at this pharmacy *(Please tick all that apply)*

- ▢ The sale of urinary incontinence related products (1)
- ▢ The sale of bowel incontinence related products (2)
- ▢ Advising on incontinence products (3)
- ▢ Dispensing/supplying medicines prescribed for the management of incontinence (4)
- ▢ Reviewing current medication that may be contributing to incontinence symptoms (5)
- ▢ Prescribing medicines for the management of incontinence (6)
- ▢ Providing appropriate information to patients/customers/carers related to incontinence and its management (e.g. leaflets, lifestyle advice) (7)
- ▢ Having a one-to-one private consultation with a patient or carer on incontinence management (8)
- ▢ Signposting to appropriate specialist continence services (9)
- ▢ Making formal referrals to appropriate specialist continence services (10)
- ▢ Providing any aspect of continence care or continence information is not part of my role (11)
- ▢ Other (12)

Q18 Q5a. Please provide details if 'other'.

____

Q20 Q6. Which of the following continence-related services are **provided at this pharmacy**:

	Yes (1)	No, but would be willing to provide in future (2)	No, and would not be willing to provide in future (3)	Unsure (4)
The sale of urinary incontinence related products (1)	○	○	○	○
The sale of bowel incontinence related products (2)	○	○	○	○
Advising on incontinence products (3)	○	○	○	○
Dispensing medicines prescribed for the management of incontinence (4)	○	○	○	○
Reviewing current medication that may be contributing to incontinence symptoms (5)	○	○	○	○
Prescribing medicines for the management of incontinence (6)	○	○	○	○
Providing appropriate information to patients/customers/carers related to incontinence and its management (e.g. leaflets, lifestyle advice) (7)	○	○	○	○
Having a one-to-one private consultation with a patient or carer on incontinence management (8)	○	○	○	○
Signposting to appropriate specialist continence services (9)	○	○	○	○
Making formal referrals to appropriate specialist continence services (10)	○	○	○	○

Advising on caffeine reduction for incontinence purposes (11)	○	○	○	○
Advising on fluid management for incontinence purposes (12)	○	○	○	○
Advising on weight loss management for incontinence purposes (13)	○	○	○	○
Advising on dietary factors for incontinence purposes (14)	○	○	○	○
Advising on bowel habits for incontinence purposes (15)	○	○	○	○
Advising on skin care for problems resulting from incontinence (16)	○	○	○	○
Advising on bladder re-training (for urgency or mixed urinary incontinence) (17)	○	○	○	○
Advising on pelvic floor muscle training (18)	○	○	○	○
Requesting patient to complete a bladder diary (19)	○	○	○	○
Administering a patient completed questionnaire to assess incontinence symptoms (20)	○	○	○	○
Other (21)	○	○	○	○

Q19 Q6a. Please provide details if 'other'.

____

Q21 Q7. How **confident would you be** in carrying out the following continence-related tasks:

	Very confident (1)	Confident (2)	Unsure (3)	Not confident (4)	Not confident at all (5)
The sale of urinary incontinence related products (1)	○	○	○	○	○
The sale of bowel incontinence related products (2)	○	○	○	○	○
Advising on incontinence products (3)	○	○	○	○	○
Dispensing medicines prescribed for the management of incontinence (4)	○	○	○	○	○
Reviewing current medication that may be contributing to incontinence symptoms (5)	○	○	○	○	○
Prescribing medicines for the management of incontinence (6)	○	○	○	○	○
Providing appropriate information to patients/customers/carers related to incontinence and its management (e.g. leaflets, lifestyle advice) (7)	○	○	○	○	○
Having a one-to-one private consultation with a patient or carer on incontinence management (8)	○	○	○	○	○
Signposting to appropriate specialist continence services (9)	○	○	○	○	○
Making formal referrals to appropriate specialist continence services (10)	○	○	○	○	○

Advising on caffeine reduction for incontinence purposes (11)	○	○	○	○	○
Advising on fluid management for incontinence purposes (12)	○	○	○	○	○
Advising on weight loss management for incontinence purposes (13)	○	○	○	○	○
Advising on dietary factors for incontinence purposes (14)	○	○	○	○	○
Advising on bowel habits for incontinence purposes (15)	○	○	○	○	○
Advising on skin care for problems resulting from incontinence (16)	○	○	○	○	○
Advising on bladder re-training (for urgency or mixed urinary incontinence) (17)	○	○	○	○	○
Advising on pelvic floor muscle training (18)	○	○	○	○	○
Requesting patient to complete a bladder diary (19)	○	○	○	○	○
Administering a patient completed questionnaire to assess incontinence symptoms (20)	○	○	○	○	○

Q22 **Section 4: Perspectives on providing continence management services**

Q23 Q8. Please select the option that best suits your opinion of each statement:

	Strongly agree (1)	Agree (2)	Unsure (3)	Disagree (4)	Strongly disagree (5)
I know how to manage patients who present with urinary incontinence (1)	○	○	○	○	○
I know how to manage patients who present with bowel incontinence (2)	○	○	○	○	○
I am confident talking to patients about urinary incontinence (3)	○	○	○	○	○
I am confident talking to patients about bowel incontinence (4)	○	○	○	○	○
I have enough knowledge of which health conditions are associated with a risk of incontinence problems at different ages (5)	○	○	○	○	○
I know where to refer patients for	○	○	○	○	○

specialist continence advice (6)					
I know where to find online resources regarding continence management (7)	○	○	○	○	○
I understand the possible impact on quality of life of incontinence on patients (8)	○	○	○	○	○
Continence management services are the responsibility of other (non-pharmacy) NHS services (e.g. GP, continence advisors, specialist hospital services) (9)	○	○	○	○	○
Continence care is a low priority in this community pharmacy (10)	○	○	○	○	○
The provision of advice on continence products is sufficient in this community pharmacy (11)	○	○	○	○	○

The provision of advice on prescription medicine for continence problems is sufficient in this community pharmacy (12)	○	○	○	○	○
There is a need for more continence management services for patients who present at this community pharmacy (13)	○	○	○	○	○
I would like to do more for patients with incontinence who attend this community pharmacy (14)	○	○	○	○	○
Providing continence management services enhances my job satisfaction (15)	○	○	○	○	○

Q24 Q9. This survey is being conducted to explore possibilities to improve accessibility to continence care and possible funding for management within community pharmacies. Your opinions are vital to understand what types of support this community pharmacy 'must have',

'should have', 'could have' or 'would not require' to be able to provide more for people with bladder and bowel leakage. Please tick an option for each type of support:

	Must have (1)	Should have (2)	Could have (3)	Would not require (4)
Increased time (1)	○	○	○	○
Sufficient staffing (2)	○	○	○	○
Appropriately trained staff (9)	○	○	○	○
Supporting resources e.g. leaflets, care records (3)	○	○	○	○
Private consultation room (4)	○	○	○	○
Training for incontinence management (5)	○	○	○	○
Funding or reimbursement (6)	○	○	○	○
Connections/collaborations for referral to specialist continence services (7)	○	○	○	○
Other (please specify) (8)	○	○	○	○

Q25 Q9a. Please provide details if 'other'.

____

Q26 Q10. We appreciate there may be difficulties to improving accessibility to continence care and management within community pharmacies. Please indicate how likely you think following issues could be a barrier to expanding community pharmacy continence support:

	Likely barrier (1)	Unsure (6)	Unlikely barrier (3)
Provision of adequate follow-up with patients (1)	○	○	○
Demand for continence management services in the local area (2)	○	○	○
Embarrassment of patients to speak about their continence-related problems in a community pharmacy (3)	○	○	○
Patient awareness that pharmacists could provide additional advice (other than selling products) about their incontinence problems (4)	○	○	○
Motivation from patients to engage with continence management services (5)	○	○	○
Enthusiasm of pharmacy staff to provide continence management services (6)	○	○	○
Impact on income to the pharmacy business (7)	○	○	○
Other (please specify) (8)	○	○	○

Q28 Q10a. Please provide details if 'other'.

____

Q29 **Section 5: Previous continence management training and interest in further training**

Q30 Q11. Have you received any training in the management of incontinence?

○ Yes (You will be taken to questions about incontinence training) (1)

○ No (You will skip the questions about incontinence training) (2)

Display This Question:

If Q11. Have you received any training in the management of incontinence? = Yes (You will be taken to questions about incontinence training)

Q31 Q12. What was the source of continence training received? *(Please tick all that apply)*

▢ Undergraduate Pharmacy Degree (1)

▢ Post-graduate Degree (2)

▢ Commercial product providers (3)

▢ Pharmaceutical companies (4)

▢ Centre for Pharmacy Post Graduate Education (or equivalent) (5)

▢ Other (please specify) (6)

Display This Question:

If Q11. Have you received any training in the management of incontinence? = Yes (You will be taken to questions about incontinence training)

Q32 Q12a. Please provide details if 'other'.

____

Display This Question:

If Q11. Have you received any training in the management of incontinence? = Yes (You will be taken to questions about incontinence training)

Q34 Q13. When was your most recent training for continence management?

- ◯ Within the last month (1)
- ◯ Between one and six months ago (2)
- ◯ Between six months and a year ago (3)
- ◯ More than one year ago (4)

Display This Question:

If Q11. Have you received any training in the management of incontinence? = Yes (You will be taken to questions about incontinence training)

Q35 Q14. How interested would you be in undertaking training in continence management in the future?

- ◯ Very interested (1)
- ◯ Interested (2)
- ◯ Neutral (3)
- ◯ Not interested (4)

Display This Question:

If Q11. Have you received any training in the management of incontinence? = Yes (You will be taken to questions about incontinence training)

Q36 Q15. How would you prefer to receive training for continence management if provided?

- ○ Online live (1)
- ○ Online recording when convenient for you (2)
- ○ In-person at training venue (3)
- ○ In-person within your community pharmacy (4)
- ○ A combination of the above formats (6)
- ○ Other (please specify) (7)

Display This Question:

If Q11. Have you received any training in the management of incontinence? = Yes (You will be taken to questions about incontinence training)

Q37 Q15a. Please provide details if 'other'.

____

Q38 **Section 6: Respondent and community pharmacy characteristics**

Q39 Q16. What is your gender?

- Male (1)
- Female (2)
- Other (3)
- Prefer not to say (4)

Q40 Q17. What age range are you in?

- 18 to 29 (1)
- 30 to 39 (2)
- 40 to 49 (3)
- 50 to 59 (4)
- 60 to 65 (5)
- 66 and older (6)

Q53 Q.18 Please select the option which best describes your ethnicity.

- White (1)
- Mixed/Multiple ethnic groups (2)
- Asian/Asian British (3)
- Black/ African/Caribbean/Black British (4)
- Other ethnic group (5)

Q41 Q19. What is your role in this pharmacy?

- ◯ Owner Pharmacist (1)
- ◯ Superintendent Pharmacist (2)
- ◯ Employee Pharmacist (3)
- ◯ Pre-registration Pharmacist (4)
- ◯ Locum Pharmacist (5)
- ◯ Pharmacy Technician (6)
- ◯ Pharmacy Assistant/Medicines Counter Assistant (7)
- ◯ Dispensing staff (9)
- ◯ Other (please specify) (10)

Q42 Q19a. Please provide details if 'other'.

____

---

*

Q43 Q20. What year did you register as a Pharmacist/Pharmacy Technician (if applicable)?
Please enter the year.

____

Q44 Q21. Which of the following qualifications do you have (if any)? *Please tick all that apply.*

- ◯ BSc/BPharm (1)
- ◯ MPharm (2)
- ◯ MSc/MPhil (3)
- ◯ PhD (4)
- ◯ Independent Prescriber (5)
- ◯ Accredited Pharmacy Technician Course (6)
- ◯ Accredited Dispensing Assistant Course (7)
- ◯ Accredited Pharmacy Medicine Counter Assistant Course (8)
- ◯ Other (please specify) (9)

Q45 Q21a. Please provide details if 'other'.

____

Q46 Q22. Which of the following best describes this community pharmacy?

- ◯ Single owner, independent, single outlet (1)
- ◯ Single owner, independent, small multiple (2-5 outlets) (2)
- ◯ Single owner, independent, large multiple (6+ outlets) (3)
- ◯ Partnership, independent, single outlet (5)
- ◯ Partnership, independent, small multiple (2-5 outlets) (6)
- ◯ Partnership, independent, large multiple (6+ outlets) (7)
- ◯ Other (please specify) (8)

Q47 Q22a. Please provide details if 'other'.

____

Q48 Q23. Please provide the first part of the postcode used by this community pharmacy (three or four characters depending on area). The pharmacy will remain anonymous, this data is to tell us about the geographical distribution of survey participants.

____

Q50 If you have any additional comments or information about the topics addressed in this questionnaire, please write them here. We are keen to hear as much about activity in this area as possible, thank you.

____

____

____

____

____

Q52 We will be conducting follow-up interviews and focus groups to gather more in-depth perspectives on continence promotion in community pharmacies within the PRIME project. If you would like to be contacted about potential involvement in future research, please add your name and contact email below. Please note, this information will only be used for this purpose and will not be shared with any third party or connected with the survey results.

Q51 Contact name

____

Q53 Contact email address

____

Q54 Section 7: Thank you for your participation

Thank you wholeheartedly for your participation in this survey. The information you have shared will be valuable in understanding the possibilities and barriers for improved accessibility to continence promotion in the community pharmacy setting.

If you would like more information about Bladder and Bowel Continence, resources are available at the following websites:

- https://www.nhs.uk/conditions/urinary-incontinence/
- https://www.nhs.uk/conditions/bowel-incontinence/
- https://www.bladderandbowel.org/

If you would like more information about the study, or about other parts of our research, please check out our project website: www.primeproject.uk or contact the study lead, Professor Nikki Cotterill.

Also, if you have been adversely affected or distressed in any way by taking part in this study, then please contact Professor Nikki Cotterill at nikki.cotterill@uwe.ac.uk

The PRIME team Thanks you for your time.

Q49 For your community pharmacy to be included in the prize draw for two £50 prizes, please follow this link to provide contact details. This will enable us to keep the study data anonymised and separately let the winning study participant know they have won.

https://uwe.eu.qualtrics.com/jfe/preview/SV_3PLVsEIknxBmanI?Q_CHL=preview&Q_SurveyVersionID=current[This will be reduced to a short URL when the survey is published]

End of Block: Default Question Block

Q14 - Q3. Where is most of your continence-related activity conducted currently?

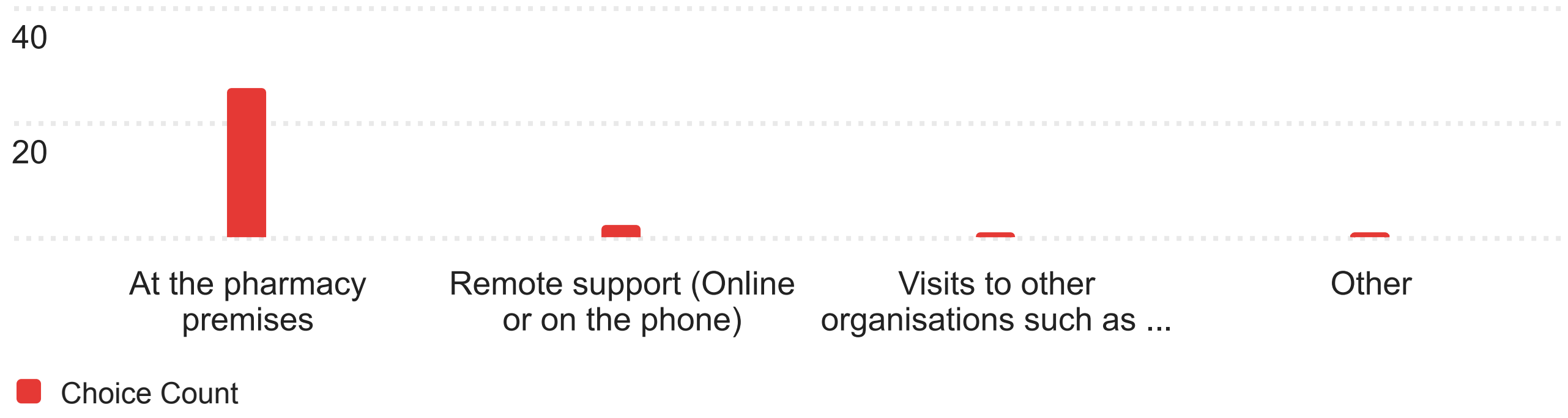

Q20 - Q6. Which of the following continence-related services are provided at this...

Field	Min	Max	Mean	Standard Deviation	Variance	Responses	Sum
The sale of urinary incontinence related products	1.00	4.00	1.24	0.73	0.53	29	36.00
The sale of bowel incontinence related products	1.00	4.00	1.93	1.05	1.10	29	56.00
Advising on incontinence products	1.00	4.00	1.24	0.77	0.60	29	36.00
Dispensing medicines prescribed for the management of incontinence	1.00	4.00	1.32	0.76	0.58	28	37.00
Reviewing current medication that may be contributing to incontinence symptoms	1.00	4.00	1.43	0.73	0.53	28	40.00
Prescribing medicines for the management of incontinence	1.00	4.00	2.19	0.86	0.74	27	59.00
Providing appropriate information to patients/customers/carers related to incontinence and its management (e.g. leaflets, lifestyle advice)	1.00	4.00	1.34	0.71	0.50	29	39.00
Having a one-to-one private consultation with a patient or carer on incontinence management	1.00	4.00	1.48	0.77	0.59	29	43.00
Signposting to appropriate specialist continence services	1.00	4.00	1.45	0.85	0.73	29	42.00

Making formal referrals to appropriate specialist continence services	1.00	4.00	1.93	0.69	0.48	29	56.00
Advising on caffeine reduction for incontinence purposes	1.00	4.00	1.54	0.98	0.96	28	43.00
Advising on fluid management for incontinence purposes	1.00	4.00	1.41	0.81	0.66	29	41.00
Advising on weight loss management for incontinence purposes	1.00	4.00	1.82	0.93	0.86	28	51.00
Advising on dietary factors for incontinence purposes	1.00	4.00	1.68	0.97	0.93	28	47.00
Advising on bowel habits for incontinence purposes	1.00	4.00	1.64	0.93	0.87	28	46.00
Advising on skin care for problems resulting from incontinence	1.00	4.00	1.57	0.78	0.60	28	44.00
Advising on bladder re-training (for urgency or mixed urinary incontinence)	1.00	4.00	1.86	0.78	0.60	29	54.00
Advising on pelvic floor muscle training	1.00	4.00	1.96	0.82	0.68	28	55.00
Requesting patient to complete a bladder diary	1.00	4.00	2.00	0.69	0.48	29	58.00
Administering a patient completed questionnaire to assess incontinence symptoms	1.00	4.00	2.00	0.64	0.41	29	58.00
Other	1.00	4.00	3.00	1.03	1.06	17	51.00

Q21 - Q7. How confident would you be in carrying out the following continence-rel...

Field	Min	Max	Mean	Standard Deviation	Variance	Responses	Sum
The sale of urinary incontinence related products	1.00	5.00	1.79	1.00	0.99	29	52.00
The sale of bowel incontinence related products	1.00	5.00	2.66	1.24	1.54	29	77.00
Advising on incontinence products	1.00	5.00	2.00	1.08	1.17	29	58.00

Dispensing medicines prescribed for the management of incontinence	1.00	5.00	1.79	1.21	1.45	28	50.00
Reviewing current medication that may be contributing to incontinence symptoms	1.00	5.00	2.14	1.06	1.12	28	60.00
Prescribing medicines for the management of incontinence	1.00	5.00	3.18	1.14	1.29	28	89.00
Providing appropriate information to patients/customers/carers related to incontinence and its management (e.g. leaflets, lifestyle advice)	1.00	5.00	2.14	1.04	1.08	29	62.00
Having a one-to-one private consultation with a patient or carer on incontinence management	1.00	5.00	2.34	1.09	1.19	29	68.00
Signposting to appropriate specialist continence services	1.00	5.00	2.34	1.15	1.33	29	68.00
Making formal referrals to appropriate specialist continence services	1.00	5.00	2.97	1.10	1.21	29	86.00
Advising on caffeine reduction for incontinence purposes	1.00	5.00	2.25	1.15	1.33	28	63.00
Advising on fluid management for incontinence purposes	1.00	5.00	2.24	1.07	1.15	29	65.00
Advising on weight loss management for incontinence purposes	1.00	5.00	2.50	1.12	1.25	28	70.00
Advising on dietary factors for incontinence purposes	1.00	5.00	2.45	1.13	1.28	29	71.00
Advising on bowel habits for incontinence purposes	1.00	5.00	2.66	1.12	1.26	29	77.00
Advising on skin care for problems resulting from incontinence	1.00	5.00	2.50	1.05	1.11	28	70.00
Advising on bladder re-training (for urgency or mixed urinary incontinence)	1.00	5.00	2.97	1.13	1.27	29	86.00
Advising on pelvic floor muscle training	1.00	5.00	2.89	1.21	1.45	28	81.00
Requesting patient to complete a bladder diary	1.00	5.00	2.79	1.06	1.13	29	81.00

Administering a patient completed questionnaire to assess incontinence symptoms	1.00	5.00	2.52	0.93	0.87	29	73.00

Q23 - Q8. Please select the option that best suits your opinion of each statement...

Field	Min	Max	Mean	Standard Deviation	Variance	Responses	Sum
I know how to manage patients who present with urinary incontinence	1.00	5.00	2.34	0.99	0.98	29	68.00
I know how to manage patients who present with bowel incontinence	1.00	5.00	2.69	1.09	1.18	29	78.00
I am confident talking to patients about urinary incontinence	1.00	5.00	2.07	0.91	0.82	29	60.00
I am confident talking to patients about bowel incontinence	1.00	5.00	2.62	1.10	1.20	29	76.00
I have enough knowledge of which health conditions are associated with a risk of incontinence problems at different ages	1.00	5.00	2.48	1.10	1.22	29	72.00
I know where to refer patients for specialist continence advice	1.00	5.00	2.66	1.09	1.19	29	77.00
I know where to find online resources regarding continence management	1.00	5.00	2.59	1.16	1.35	29	75.00
I understand the possible impact on quality of life of incontinence on patients	1.00	5.00	1.79	0.80	0.65	29	52.00
Continence management services are the responsibility of other (non-pharmacy) NHS services (e.g. GP, continence advisors, specialist hospital services)	1.00	5.00	2.90	1.12	1.27	29	84.00
Continence care is a low priority in this community pharmacy	1.00	5.00	3.76	0.97	0.94	29	109.00
The provision of advice on continence products is sufficient in this community pharmacy	1.00	4.00	2.55	0.81	0.66	29	74.00

The provision of advice on prescription medicine for continence problems is sufficient in this community pharmacy	1.00	4.00	2.41	0.93	0.86	29	70.00
There is a need for more continence management services for patients who present at this community pharmacy	1.00	4.00	2.34	0.80	0.64	29	68.00
I would like to do more for patients with incontinence who attend this community pharmacy	1.00	5.00	2.00	0.83	0.69	29	58.00
Providing continence management services enhances my job satisfaction	1.00	5.00	2.17	0.87	0.76	29	63.00

Q24 - Q9. This survey is being conducted to explore possibilities to improve acce...

Field	Min	Max	Mean	Standard Deviation	Variance	Responses	Sum
Increased time	1.00	3.00	1.45	0.62	0.39	29	42.00
Sufficient staffing	1.00	3.00	1.34	0.54	0.29	29	39.00
Appropriately trained staff	1.00	4.00	1.39	0.72	0.52	28	39.00
Supporting resources e.g. leaflets, care records	1.00	3.00	1.41	0.62	0.38	29	41.00
Private consultation room	1.00	2.00	1.17	0.38	0.14	29	34.00
Training for incontinence management	1.00	4.00	1.38	0.67	0.44	29	40.00
Funding or reimbursement	1.00	3.00	1.29	0.52	0.28	28	36.00
Connections/collaborations for referral to specialist continence services	1.00	3.00	1.38	0.55	0.30	29	40.00
Other (please specify)	1.00	4.00	2.50	1.28	1.65	10	25.00

Q30 - Q11. Have you received any training in the management of incontinence?

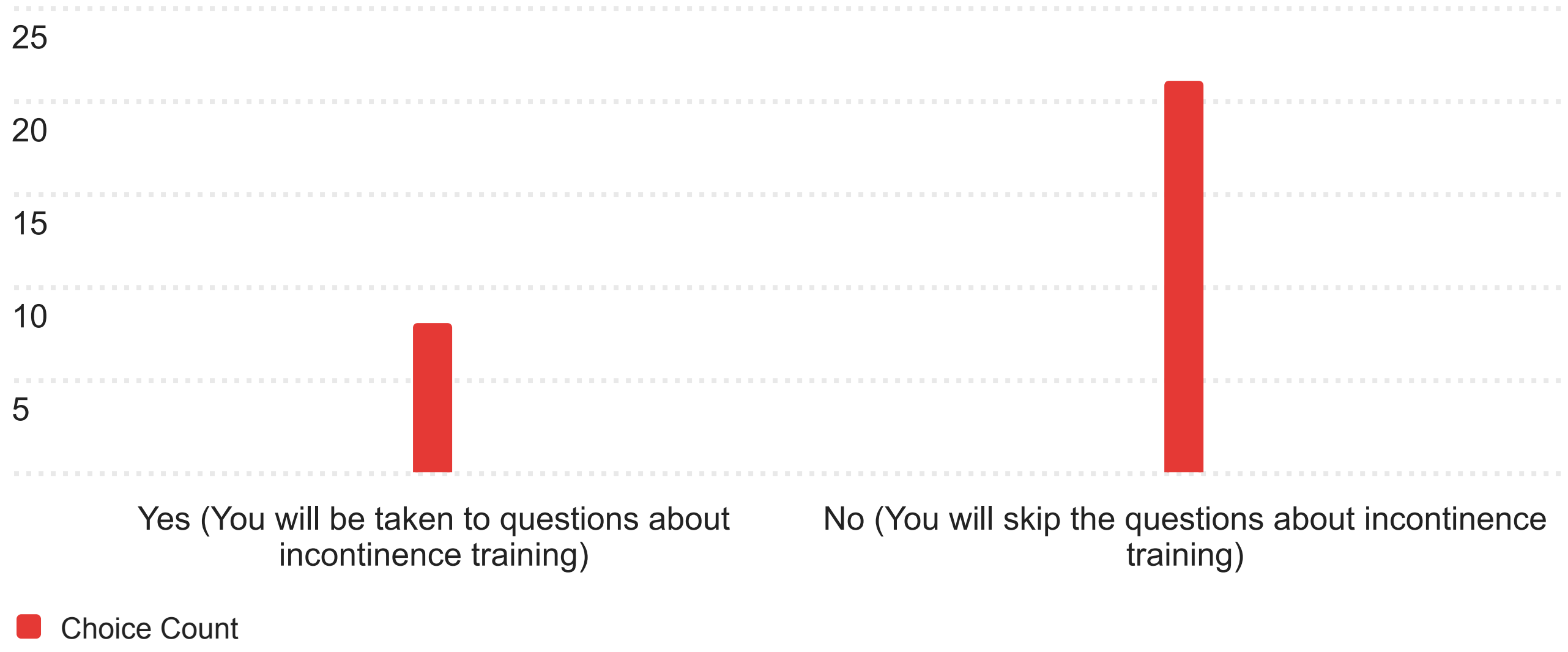

Q31 - Q12. What was the source of continence training received? (Please tick all that apply)

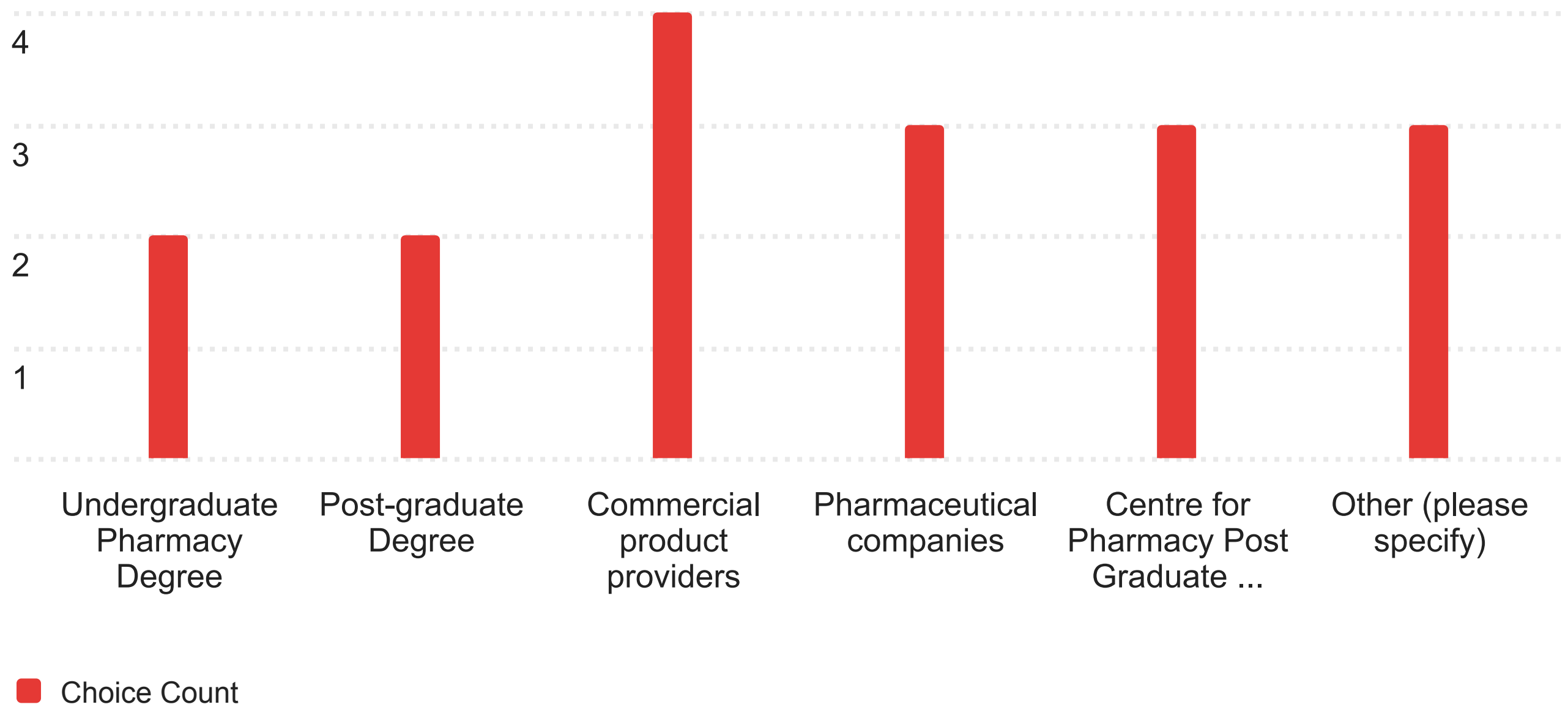

Supplement: Supplementary file 1 [file 12913_2026_15123_MOESM1_ESM.pdf]
