# Supplementary material for: Pharmacy role in the proMotion of continencE (PRIME): a co-production study to develop a pharmacy bladder and bowel service
Source: BMC Health Serv Res. 2026 Jul 24;26:1445. doi: 10.1186/s12913-026-15123-8 (PMC13689019; doi:10.1186/s12913-026-15123-8)
Supplement: Supplementary file 2 [file 12913_2026_15123_MOESM2_ESM.docx]

# Pharmacy Role In the proMotion of continencE (PRIME): a co-production study to develop a Pharmacy Bladder and Bowel Service

# Appendix 2: PRIME Co-Production Study Evidence Brief

**Pharmacy Role in the Promotion of Continence (PRIME)**

**Information Pack**

**April 2023**

**Document guidance**

The purpose of this information pack is to provide a summary of information about the topics that are relevant to this study. The pack is for all study participants including members of the public, representatives of the pharmacy profession, bladder and bowel health care providers and service commissioners. As such, the information is intended to be presented in a way that is clear for everybody involved.

This information includes the results of interviews and group discussions that have been undertaken with the above groups of people. We have also undertaken a survey of community pharmacy (chemist) staff and a review of relevant research to gather information about what is known about the topic already.

When information is presented, it might be followed by number written in superscript e.g. ^1^. This number relates the statement to where it came from, also called references. These references are listed in alphabetical order at the end of the pack. This reference list is provided for information only and there is no expectation or need for you to refer to or read the original document.

**Background**

1. **Incontinence**

***What is Incontinence?***

Incontinence is where a person is unable to stop the leakage of urine (wee) or faeces (poo), often talked about as ‘accidents’. Far more people experience these symptoms than is often realised for many and varied reasons. There are many misconceptions surrounding incontinence, but the truth is it affects around 1 in 5 adults, men and women, of any age including children. People are often reluctant to talk about incontinence due to associated embarrassment. This means less is known about who is experiencing these symptoms and also that people are often not able to get the help that can make a difference.

Throughout this project we are trying to focus on ‘promoting continence’ or enabling people to improve their symptoms as much as possible.

***What causes incontinence/continence problems?***

Incontinence can happen for many reasons, the most frequent include: following childbirth; alongside a condition that affects the brain or nervous system such as multiple sclerosis or spinal cord injury; conditions that cause an urgent need to empty the bladder or bowels such as overactive bladder; following surgery to the pelvic area such as prostate surgery; an inability to recognise the signals to go to the toilet which can occur with dementia, or an inability to reach the toilet in time due to issues moving around.

***How can incontinence problems be managed?***

There are lots of ways to address incontinence that start with a number of options that can be tried at home. We call these Conservative measures as they do not involve any medication or surgery. Conservative measures include lifestyle habits such as looking at what you eat and drink and what might trigger leakage. Pelvic floor exercises are also advised for certain types of incontinence to strengthen the muscles in the pelvic floor that can help to prevent leakage. Managing urgent symptoms can also be explored to enable people to have enough time to reach the toilet. Some of these measures can be tried without any healthcare input, while some are better with the support of healthcare advice. Importantly, if these strategies do not work, there are other options that can be discussed with specialists in this area, and knowing when to seek help is important.

***How are healthcare services for incontinence accessed?***

Help and advice for continence problems can be sought by going to the GP, pharmacy, local continence services, and through bladder and bowel helplines and charities. Normally the access point for treatment services is through GPs, who work closely with other professionals within their surgery and the wider community. GPs can do simple tests such as on blood or urine. They can discuss self-help advice and possible prescriptions for drug treatments and can refer to another health professional such as a continence advisor, physiotherapist or occupational therapist if needed. If the continence symptoms are very severe or difficult to manage, a referral to hospital specialists such as to a urologist, gastroenterologist or colorectal surgeon may be needed for further investigations or surgical options.

2. **Community pharmacy**

Community pharmacies are premises that were previously called chemists or chemist shops. For the purpose of this study, the term ‘community pharmacy’ includes pharmacies on the high street, in supermarkets, or in other locations such small, individually-owned pharmacies in small communities. There are around 11,500 community pharmacies in England, and 90% of the population lives within 20 minutes’ walk of one of these healthcare settings^1^. Community pharmacies provide a wide range of services to people and the communities that they serve. No appointment is necessary, and most pharmacies have private consultation rooms.

***Pharmacy staff***

Pharmacies have a number of trained staff in their team who work together to deliver services.

Pharmacists are health professionals with university degrees. As with general practitioners (GPs), community pharmacists are also part of the NHS. Pharmacy students undertake a four year degree course, before undertaking a one-year placement working in a pharmacy under the supervision of an experienced pharmacist. At the end of this year they take a professional examination and if successfully completed they are able to register as a pharmacist ^2^.

Pharmacy Technicians “are professional skilled and essential members of the pharmacy team, who broadly prepare, dispense, supply and issue a wide range and variety of medicines to patients. Whilst they work under the supervision of the pharmacist, Pharmacy Technicians are responsible and accountable for their own accurate and safe practice” ^2^.

Medicine counter assistants (MCAs) are generally the first point of contact for patients and/or members of the public, and support the delivery of pharmacy services. They often undertake the prescription reception process, including supporting patients to complete the declarations on NHS prescriptions. Self-help advice and basic healthy lifestyle information and advice can be provided by MCAs under the supervision of the pharmacist ^2^.

All pharmacy staff are required to respect the confidentiality of patient information and to follow the NHS code of Practice on Confidentiality, the Common Law on confidentiality and the Data Protection Act.

***NHS Pharmacy service***

Community pharmacies in England deliver services as part of their NHS contract, similar to other providers such as general medical practices. There are ‘essential services’ that all pharmacies have to provide, such as the dispensing of medicines and medicinal products, advising patients on self-care and medication review/support following a hospital discharge ^3^.

Other more specialised services are optional and may be delivered by pharmacies on a national or local level ^3^. In England, the community pharmacy **‘advanced service’** is an optional service with specified procedures that a pharmacy can choose to provide. An example is the New Medicine Service that advises patients on the best use of a newly prescribed medicine.

“Community pharmacies can also provide **locally commissioned services** – these are commissioned by public bodies, to meet the needs of particular local populations. Examples include sexual health services, needle and syringe exchange services, or smoking cessation services.”^3^

3. **Why explore continence promotion in community pharmacy?**

Few people with continence problems seek help from health care professionals e.g. GPs, pharmacists ^4,5^. One in five women and one in 20 men who have urinary incontinence seek help , and this becomes even less likely in some cultures or with people who do not speak English, ^4^.

From our own work in the early phases of this project, we know that people with incontinence symptoms, or carers of those with symptoms, often do not know that there are options to treat incontinence or how to access services. In addition, specialised continence services can be difficult to access ^6^ leaving individuals with the only option of *“having to find their way through it alone”*.

Community pharmacies are a trusted and convenient setting for people to receive general advice on their healthcare needs. Pharmacies already will provide general advice and support on continence care as part of their NHS service, including often stocking a range of continence products. However, pharmacies could be an ideal setting for identifying and supporting people to access treatment for continence problems, in particular for those who might not seek help elsewhere such as their GP ^7^.

In terms of the need for sensitivity and privacy, a specialised pharmacy-based bladder and bowel service (PBBS) could have similarities to other local services that are provided from community pharmacies such as emergency hormonal contraception, erectile dysfunction, sexually transmitted disease diagnosis and treatment.

This is an opportunity to reach more people with continence problems who might otherwise not seek care and for whom symptom improvement is possible. A pharmacy-based service may also reduce pressure on general practices, bladder and bowel continence services, and other care providers ^8^.

4. **What have we found out through the PRIME study?**

*What is known already in the published literature*

We looked at all the relevant research that has explored how community pharmacy personnel might support people with continence problems. The key findings were:

- Pharmacy staff had generally positive attitudes towards the provision of continence interventions and services within community pharmacies.
- The introduction of a pharmacy-based continence service would probably need to be accompanied by a public awareness campaign to identify pharmacies as a place for continence care.
- An increase in pharmacist knowledge through training is likely to be important in providing staff with greater confidence for assessment and appropriate referral ^9^.
- Appropriate funding is key to the success of future pharmacy-based continence services ^10^.
- The most commonly reported barrier to the success of pharmacy services is insufficient resources, particularly workforce and time ^10,11^.

*National community pharmacy survey*

Thirty members of community pharmacy personnel completed the survey. The main findings were as follows:

- Most respondents (90%, n=27) reported that continence care is a part of their role and most (87%, n=26) were based in pharmacies that sold urinary incontinence products, while fewer (43%, n=13) sold faecal incontinence products.
- The key requirements for future continence service provision were access to a private consultation room (n=24), appropriate funding (n=21), adequate staffing (n=20) and access to training (n=20).

Figure 1 shows community pharmacist perspectives on current and possible future continence care provision. The blue columns show what is already happening in community pharmacies, the orange columns show us what community pharmacies would be prepared to provide, and the grey columns show what is felt to be beyond the scope of community pharmacies.

Community pharmacist perspectives on current and future continence provision

Number of respondents

|  | Currently provided | Not currently provided but potential | Not currently provided and not future potential |
| --- | --- | --- | --- |
| Caffeine advice | 20 | 4 | 1 |
| Fluid advice | 21 | 6 | 0 |
| Dietary advice | 16 | 8 | 1 |
| Bladder diary | 6 | 18 | 4 |
| Questionnaire assessment | 5 | 20 | 3 |
| Bladder retraining | 9 | 17 | 1 |
| Pelvic Floor Muscle Training | 8 | 15 | 3 |
| Formal referral | 7 | 18 | 3 |

*In-depth interviews*

We interviewed pharmacy staff, people using community pharmacies, bladder and bowel service staff and service commissioners. The key findings were:

- Additional training and resources will be required to support a new pharmacy bladder and bowel service. For example, information technology, private rooms, awareness raising materials supported by public awareness campaigns.
- Pharmacist and commissioner participants suggested appropriate funding and high level changes at a policy level may be required to enable pharmacies to include a new bladder and bowel service.
- Service users and bladder and bowel care staff highlighted the need for sensitivity and privacy.
- A clear framework would be required that enables community pharmacists to provide this service, that is supported by national guidance. This would include who would be included, how they should be approached, what should be offered, and how to show it works.
- The main barriers to a new service were considered to be the time staff have available, being really clear about what the service will and will not offer, and embarrassment preventing people from revealing they have symptoms.

5. **Workshop Activities**

Within the workshop we will be holding discussions around what you think about the information provided above, to gather different opinions. We will be considering what it will be like to use the service as patients or carers, and to provide the service as community pharmacy staff. We will think about what a journey will look like for somebody who might use the new Pharmacy Bladder and Bowel Service and discuss what would be needed at each stage to make it work. This will be discussed in more detail during the workshop and the plan for the session is included separately. Throughout all of these discussions there are no right or wrong answers. This is a process to think through all the different possibilities to develop a service that is fit for purpose, so all ideas are very welcome for discussion.

**References**

1.	Community pharmacies in England 2006-2022. Statista. Accessed February 10, 2023. https://www.statista.com/statistics/418071/community-pharmacies-in-england/

2.	About community pharmacy. PSNC Website. Accessed April 5, 2023. https://psnc.org.uk/pharmacy-the-heart-of-our-community/about-community-pharmacy/

3.	Baird B, Beech J. Community pharmacy explained. The King’s Fund. Published December 16, 2020. Accessed February 10, 2023. https://www.kingsfund.org.uk/publications/community-pharmacy-explained

4.	Newman D, Cockerell R, Griebling TL, Rantell A, Houten P, Palmer MH. Primary prevention, continence promotion, models of care and education. In: Abrams P, Cardozo L, Wagg A, Wein A, eds. *Incontinence: Proceedings of the Sixth International Consultation on Incontinence, Tokyo. Sixth*. Health Publications Limited; 2017:2427-2478.

5.	Duong E, Al Hamarneh YN, Tsuyuki RT, et al. Case finding for urinary incontinence and falls in older adults at community pharmacies. *Can Pharm J Rev Pharm Can*. 2019;152(4):228-233. doi:10.1177/1715163519852378

6.	East N, Council S, Metropolitan B, Council B, Council DC, Council NC. PCF Freedom of Information requests to local authorities – summary of results. Published online 2017.

7.	Duong E, Al Hamarneh YN, Tsuyuki RT, Wagg A, Hunter KF, Schulz J. Case finding for urinary incontinence and falls in older adults at community pharmacies. *Can Pharm J*. 2019;152(4):228-233.

8.	Lee F, Motta M, Wilcock M. Evaluation of a community pharmacy service for uncomplicated urinary tract infection. The Pharmaceutical Journal. Accessed December 6, 2022. https://pharmaceutical-journal.com/article/research/evaluation-of-a-community-pharmacy-service-for-uncomplicated-urinary-tract-infection

9.	Eades CE, Ferguson JS, O’Carroll RE. Public health in community pharmacy: a systematic review of pharmacist and consumer views. *BMC Public Health*. 2011;11:582. doi:10.1186/1471-2458-11-582

10.	Shoemaker SJ, Curran GM, Swan H, Teeter BS, Thomas J. Application of the Consolidated Framework for Implementation Research to community pharmacy: A framework for implementation research on pharmacy services. *Res Soc Adm Pharm RSAP*. 2017;13(5):905-913. doi:10.1016/j.sapharm.2017.06.001

11.	Weir NM, Newham R, Dunlop E, Bennie M. Factors influencing national implementation of innovations within community pharmacy: a systematic review applying the Consolidated Framework for Implementation Research. *Implement Sci*. 2019;14(1):21. doi:10.1186/s13012-019-0867-5
